# Current News

**Published:** 1891-03

**Authors:** 


### Current News.

### COMPLIMENTARY BANQUET TO THE VETERANS OF THE DENTAL PROFESSION.

THIS banquet, which attracted wide attention on account of its novelty, took place at the time appointed. The announcement was recognized as a graceful tribute to the fathers of the profession, and met with a response of generous feeling in many sections of the country.

The attendance was an unusual one, both in numbers and character, Boston, Chicago, Washington, Philadelphia, and other places furnishing representatives to do honor to the men and the occasion. The large banqueting hall was filled, one hundred and seventy-five being seated.

The following veterans accepted and were present: Dr. John B. Rich, Washington, D. C.; Dr. Jesse C. Green, West Chester, Pa.; Dr. Spencer Roberts, Philadelphia; Dr. C. A. Kingsbury, Philadelphia; Dr. J. J. Wetherbee, Boston; Dr. F. H. Burras, Long Island; Dr. W. B. Hurd, Long Island; Dr. A. D. Newell, New Jersey; Dr. J. Hayhurst, New Jersey; Dr. Jere. A. Robinson, Michigan; Dr. A. J. Volck, Baltimore; Dr. John Allen, New York; Dr. W. H. Atkinson, New York; Dr. W. H. Dwinelle, New York; Dr. S. A. Main, New York; Dr. L. S. Straw, Newburg, N. Y.

The following accepted, but were detained: Dr. Elisha G. Tucker, Boston; Dr. Ambrose Lawrence, Boston; Dr. Finley Hunt, Washington; Dr. W. H. H. Thackston, Virginia; Dr. Augustus W. Brown, New York; Dr. C. W. Spaulding, St. Louis.

The following, for various reasons, declined: Dr. Edward Maynard, Washington, D. C.; Dr. Edwin J. Dunning, Newton Centre, Mass.; Dr. J. DeHaven White, Philadelphia; Dr. Daniel Neall, Philadelphia; Dr. Edward Townsend, Philadelphia; Dr. R. C. Marshall, Maryland; Dr. J. Smith Dodge, Morristown, N. J.; Dr. A. W. Kingsley, New Jersey; Dr. J. F. Fowler, Florida; Dr. J. H. Farnsworth, Detroit; Dr. Corydon Palmer, Warren, Ohio; Dr. L. N. Bristol, Lockport, N. Y.; Dr. E. D. Fuller, Peekskill, N. Y.; Dr. B. W. Franklin, Ithaca, N. Y.; Dr. W. A. Royce, Middletown, N. Y.

Dr. Norman W. Kingsley presided, and in a pleasant, witty speech called attention to the supposed origin of the respected guests and to the fact that the ranks of these fathers in the profession were being rapidly depleted, and that it was our duty as it was our pleasure to honor them while they were still with us.

The committee had only been able to find forty who were in practice fifty years ago; but had heard of some few others since the invitations were sent out, but of this number, forty, one-half, were here to-night. He then referred to the few in Europe, and humorously alluded to the difficulties which might be encountered with the McKinley Bill in importing material that possibly might interfere with home production. He then called on Dr. Marvin, of Brooklyn, to welcome the guests.

Dr. Marvin said, "I have been invited to speak a few words to you fathers on dentistry. In obedience to an invitation, you have left your homes to partake of our hospitality and give us an opportunity to see how men look and act who have practised dentistry for half a century.

"I am but voicing the sentiment of my own heart in tendering you this earnest welcome, and in this testimony every one present is a sharer. We meet you as men of mature years, whose judgments have been proven in the many communities in which you have labored. We meet you as pioneers in a profession when its literature was weak; when its methods were crude; when nearly all that goes to make up the profession, as now understood, was absent. As veterans who have come down through all these intervening years of struggle, we welcome you.

"We welcome you for the reason that you have freely contributed all you possessed of professional knowledge for the instruction of the neophyte as well as those further advanced. We welcome you in that you have advanced the literature of the profession, and we give you more than all glad welcome and extend our congratulations that you have lived to see this happy day. And now may the sea of life flow smoothly, respected fathers, and as you have done your share to give honor to the profession, we do well to honor you. Bear with you the assurance of our earnest hope for a continued future of that usefulness so well demonstrated in the past. Let us now all rise, and with fervor respond to the sentiment, 'Our Fathers in Dentistry.'"

The President then dealt humorously with the supposed antiquity of the guests, and called on Dr. Hurd, of Brooklyn, N. Y., whose vigorous tones and mental quickness gave no signs of loss of power. His speech in response to that portion of the President's remarks in relation to the Ark was most excellently done, and called forth the plaudits in unreserved measure. Having disposed of the worthy presiding officer, he said,—

"Brethren, time has changed us, and this is more apparent to others than to ourselves, as we cordially greet each other in the old familiar tones. We met often in the past when the fires burned more brightly than now; but I know you are not willing to say or to feel that you are only burnt embers.

"To-night we have been highly honored. We have had good things said to us through the eloquent lips of Dr. Marvin. The proper man was selected to give the veterans the right hand of welcome. How applicable to him, it seemed to me, were the words of the dying girl,—'I am almost there, mother.'

"To you, young men, let me say, I wish you to get all the good possible from your profession. It is a noble one. Build your castles while in your youth, and we, the old men of the profession, will reflect and rejoice that the day's work is almost done."

It would be pleasant to give full abstracts from the remarks of the other speakers, but space will not permit further extension.

Dr. F. H. Burras spoke of his early experiences, and was followed by Dr. Rich, of Washington, formerly of New York, who paid a just and glowing tribute to the work of Dr. Hayden. Probably no other man present could speak of these earlier pioneers from more thorough personal knowledge, and his remarks bridged over the present to the past, and vividly pictured the men of the transition stage from empiricism to professional worth.

Dr. Sheppard, of Boston, made some interesting remarks on the life-work of Dr. Tucker, of Boston, who, though eighty-three years of age, has a clientele the very best, and performs his labor with a hand as sure and eyesight unimpaired as thirty years ago.

Dr. C. A. Kingsbury, of Philadelphia; Dr. J. J. Wetherbee, of Boston; Dr. W. H. Dwinelle and Dr. John Allen, of New York, and Dr. S. S. Straw, of Newburg, N. Y., made appropriate and interesting remarks.

Letters were read from Dr. J. Smith Dodge, Dr. Edward Maynard, Dr. Edwin J. Dunning, Dr. C. W. Spaulding, Dr. Elisha G. Tucker, and Dr. R. C. Mackall.

A telegram was received from Chicago, presenting compliments to the "Old Guard" and regrets at not being able to be present.

At the President's request, the company arose and sang "Auld Lang Syne," and, with good wishes for the veterans, departed for their several homes, carrying with them the memory of a pleasant reunion.

---

PITTSBURG, PA.

The annual meeting of the Odontographic Society convened in the parlor of Library Hall, January 22, 1891. The following officers were elected for the ensuing year: President, F. S. Whitslar; First Vice-President, W. F. Fundenberg; Second Vice-President, W. A. Kessler; Secretary, W. H. Fundenberg; Treasurer, W. A. Lee; Librarian, H. L. Reinecke.

*Censors.*—J. S. Goshorn, C. J. Phillips, L. Depuy.

*Executive Committee.*—W. F. Fundenberg, George Shidle, C. B. Bratt.

